# Precisely printable and biocompatible silk fibroin bioink for digital light processing 3D printing

**DOI:** 10.1038/s41467-018-03759-y

**Published:** 2018-04-24

**Authors:** Soon Hee Kim, Yeung Kyu Yeon, Jung Min Lee, Janet Ren Chao, Young Jin Lee, Ye Been Seo, Md. Tipu Sultan, Ok Joo Lee, Ji Seung Lee, Sung-il Yoon, In-Sun Hong, Gilson Khang, Sang Jin Lee, James J. Yoo, Chan Hum Park

**Affiliations:** 1 Nano-Bio Regenerative Medical Institute, College of Medicine, Hallym University, Chuncheon, 24252 Republic of Korea; 2 School of Medicine, George Washington University, Washington, D.C. 20037 USA; 3 Division of Biomedical Convergence, College of Biomedical Science, Kangwon National University, Chuncheon, 24341 Republic of Korea; 4 Department of Molecular Medicine, School of Medicine, Gachon University, Incheon, 406-840 Republic of Korea; 5 Department of BIN Convergence Technology, Department of Polymer Nano Science & Technology and Polymer Materials Fusion Research Center, Chonbuk National University, Jeonju, 54896 Republic of Korea; 6 Wake Forest Institute for Regenerative Medicine, Wake Forest School of Medicine, Medical Center Boulevard, Winston-Salem, NC 27157 USA; 7 Departments of Otorhinolaryngology-Head and Neck Surgery, Chuncheon Sacred Heart Hospital, School of Medicine, Hallym University, Chuncheon, 24252 Republic of Korea

## Abstract

Although three-dimensional (3D) bioprinting technology has gained much attention in the field of tissue engineering, there are still several significant engineering challenges to overcome, including lack of bioink with biocompatibility and printability. Here, we show a bioink created from silk fibroin (SF) for digital light processing (DLP) 3D bioprinting in tissue engineering applications. The SF-based bioink (Sil-MA) was produced by a methacrylation process using glycidyl methacrylate (GMA) during the fabrication of SF solution. The mechanical and rheological properties of Sil-MA hydrogel proved to be outstanding in experimental testing and can be modulated by varying the Sil-MA contents. This Sil-MA bioink allowed us to build highly complex organ structures, including the heart, vessel, brain, trachea and ear with excellent structural stability and reliable biocompatibility. Sil-MA bioink is well-suited for use in DLP printing process and could be applied to tissue and organ engineering depending on the specific biological requirements.

## Abstract (web summary)

Although 3D bioprinting technology has gained much attention in the field of tissue engineering, there are still several significant challenges that need to be overcome. Here, the authors present silk fibroin bioink with printability and biocompatibility suited for digital light processing 3D printing.

## Introduction

Recently, bioprinting technology has moved toward the goal to create more complex structures with different tissue components and intrinsic microvasculature^1,2^. Bioprinting permits cells, biomaterials, and bioactive molecules to be placed in a precise manner, so as to create a complex three-dimensional (3D) tissue structure for biological and clinical applications^3,4^. Bioprinting technologies can be classified^3–5^ into inkjet printing, extrusion printing (fused deposition modeling; FDM), light-assisted bioprinting^6–11^, including digital light processing (DLP) and laser-based printing (Table 1). Inkjet bioprinting that is similar to conventional two-dimensional inkjet printing has the advantages of being relatively low cost and capable of moderate printing speed (mm s^−1^); however, disadvantages include the inability to use high-viscosity materials and high cell density due to nozzle clogging and inability to construct 3D tissue structures^12^. The extrusion bioprinter was developed by modifying the inkjet printer and uses an air pump or a screw plunger to dispense bioinks. Because of this design, the extrusion type printer is compatible with hydrogels of various viscosities, but larger mechanical stresses on the encapsulated cells from more viscous hydrogels and a relatively long printing time can reduce cell viability by 40–80%^3,13^. In contrast, DLP bioprinter can overcome these limitations. They create models in a layer-by-layer fashion unlike other printing modalities through photopolymerization by ultraviolet (UV) light. As a result, DLP achieves high resolution (about 1 μm) and with rapid printing speed (~30 min, mm^3^ s^−1^) regardless of the layer’s complexity and area. In addition, DLP printing increases cell viability beyond 85–95% due to the short printing time and nozzle-free printing technique^5,14,15^.

**Table 1:** Recent works on light-assisted bioprinting

Printer	Bioink	Photoinitiator	Light source	Mechanical properties	Target tissue or structure	Cell type	Culture	Ref.
DLP	GelMA, GMHA	LAP	UV (365 nm)	>4 kPa (stiffness)	Liver tissue	hiPSC-HPCs	In vitro (10 days)	^6^
STL	PEGDA and GelMA	Eosin Y-based photo initiator	Customized visible light	Young’s modulus (60 kPa)	Hydrogel	NIH 3T3 cells	In vitro (5 days)	^7^
LAB	HA and collagen	NA	Pulsed laser	NA	Bone tissue regeneration	Mesenchyma stroma cells	In vitro (8 days), in vivo (42 days)	^8^
STL	Methacrylated PEG-co-PDP	LAP	Visible light projection SLA (3200 lumens)	38 kPa (stiffness)	Cell-laden hydrogel	HUVEC cells	In vitro (10 days)	^10^

*LAP* lithium phenyl-2,4,6 trimethylbenzoyl phosphinate, *GelMA* gelatin methacrylate, *GMHA* glycidyl metacrylate hyaluronic acid, *PEGDA* polyethylene glycol diacrylate, *HA* hyaluronic acid, *PEG-co-PDP* poly(ethylene glycol-co-depsipeptide), *hiPSC-HPC* human iPSC-derived hepatic progenitor cells, *DLP* digital light processing based bioprinter, *STL* stereolithography, *LAB* laser-assisted bioprinter

The printable materials or bioinks need to satisfy several essential criteria in terms of printability, biocompatibility, and biomimetic properties, including structural and mechanical stability. All of these requirements are essential for long-term shape constancy^7,16^. Particularly, when using the DLP modality, the bioink must be able to deposit in a layer-by-layer fashion, create Z-layer definition, and be photocurable^5^.

Hydrogels, which form 3D crosslinked hydrated fibers, are suitable as a bioink in 3D bioprinting. They can be used as a cell matrix, and provide a mechanically supportive microenvironment, that can be modified to mimic native tissue and its extracellular matrix. There are only a few reported biomaterials capable of producing a hydrogel for 3D bioprinting, including fibrinogen, agarose, gelatin, hyaluronic acid, and alginate^13,17^. A mixture of chitosan and polyethylene glycol diacrylate (PEGDA)^18^, a mixture of pluronic diacrylate and hyaluronic acid methacrylate^19^, and a mixture of PEGDA and gelatin methacrylate (GelMA) hydrogel^7^ have been reported as potential bioinks for DLP printing. However, the hydrogels based on synthetic materials have inherently low cell adhesion abilities, and the natural material-based hydrogels have insufficient stiffness, thereby making it difficult to control the matrix’s rigidity^20^. In DLP printing, the dynamics of the polymerization can be adjusted by changing the power of the light source, the printing rate, and the type and concentrations of the photoinitiators, however, ultimately, the biomaterial itself controls the printability and mechanical properties of the printed materials.

Silk fibroin (SF), a natural fibrous protein produced by *Bombyx mori*, has been used for a variety of biomedical and biotechnological applications, including as a wound dressing^21,22^, enzyme immobilization matrix^23^, vascular prosthesis, and structural implant^24,25^. SF can be processed into different forms and structures, including as a film, gel, membrane, powder, and porous sponges^25–27^ under all-aqueous processing conditions and has been applied to tissue engineering. For other purposes, SF has been incorporated with various materials^28^ through chemical modifications such as coupling reactions, amino-acid modifications, and grafting reactions depending on the particular application. Addition of methacrylate groups to the amine-containing side groups of a material can be used to make it light polymerizable into a hydrogel. A material’s degradation time can also be tailored by varying the degree and location of methacrylation^29–31^. We hypothesized that SF would be an excellent bioink for DLP 3D bioprinting through methacrylation. Until now, SF itself has not been used for DLP printing due to the absence of a crosslinkage site essential for photopolymerization and we do not believe that SF has ever been modified with methacrylate groups directly for light polymerization.

In this study, we demonstrate a technique to develop an effective bioink for DLP printing with chemically modified SF by glycidyl methacrylate (GMA) (Sil-MA). We evaluate the degree of methacrylation on SF modified by various GMA amounts and characterize its physical and mechanical properties depending on Sil-MA concentration relative to the potential for suturing. Furthermore, we demonstrate the biocompatibility of the Sil-MA hydrogels prepared by DLP printing and its printability for different organs with complex structures.

## Results

### Sil-MA characterization

**Fig. 1:**
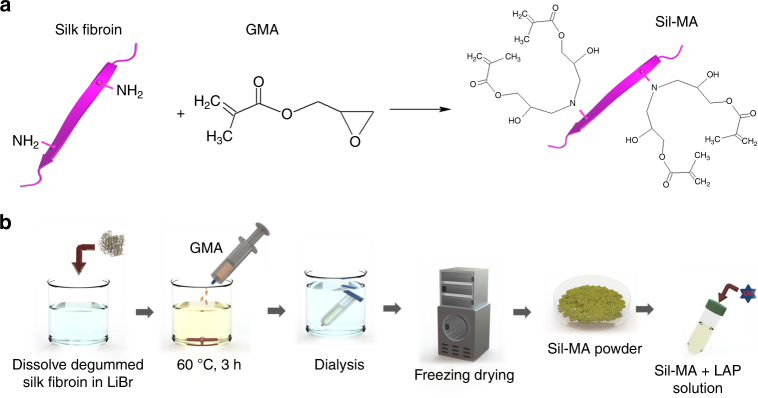
Fabrication of chemically modified silk fibroin (SF) by glycidyl methacrylate (GMA) (Sil-MA) as pre-hydrogel. **a** Modification of SF molecule with GMA. SF is covalently immobilized with GMA, which is a donor of vinyl double bond as a UV-crosslinking site. **b** Schematic representation for methacrylation of SF. Degummed silk was dissolved in 9.5 M LiBr LiBr and GMA was dropped into the solution with stirring for 3 h at 60 °C. It was dialyzed to remove salts in distilled water at room temperature for 4 days and then freeze-dried. Lithium phenyl(2,4,6-trimethylbenzoyl) phosphinate (LAP) was added to GMA-modified SF solution

**Fig. 2:**
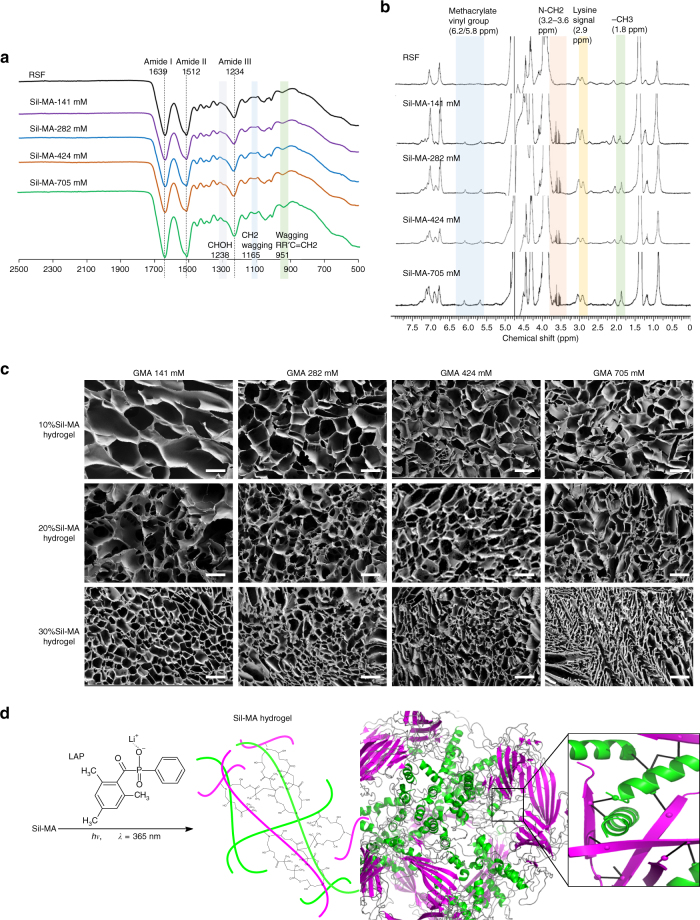
Characterization of Sil-MA pre-hydrogel and Sil-MA hydrogel depending on methacrylation degrees. **a** FT-IR spectra and **b** ^1^H-NMR spectra of unsubstituted SF and Sil-MA. In the FT-IR spectra, amide I (1639 cm^−1^), amide II (1512 cm^−1^), and amide III (1234 cm^−1^) shown in β-sheet of SF and spectra related with GMA such as CHOH, RR′C=CH_2_ were found. The modification of lysine residues in SF with the increase of GMA was confirmed by the gradual decrease in the lysine signal at *δ* = 2.9 ppm and the increase in the methacrylate vinyl group signal at *δ* = 6.2–6 and 5.8–5.6 ppm, and the methyl group signal at *δ* = 1.8 ppm. **c** FE-SEM images of Sil-MA hydrogels, presenting the effect of the degree of methacrylation and Sil-MA contents on the pore sizes of Sil-MA hydrogels. Scale bar represents 100 μm. **d** Reaction process for the photopolymerization of Sil-MA in the presence of LAP photoinitiator and 3D structure of Sil-MA hydrogel formed via digital light process (DLP) printing. With the presence of photoinitiator, the vinyl double bonds on GMA could react with each other intra-chain or between chains. SF chains themselves could entangle each other during photopolymerization. Green and magenta colors indicate alpha helix and beta sheet motif in the secondary structure of SF, respectively

The Sil-MA was synthesized by methacrylate substitution of the primary amines of SF (Fig. 1a). To find suitable GMA amount, Sil-MA was fabricated by adding GMA with various molar ratios from 141 to 705 mM into SF solution (Fig. 1b). The resulting Sil-MA and Sil-MA hydrogel were characterized depending on the different GMA amounts. Modification of GMA on SF was confirmed through identification of GMA-related peaks and SF-related peaks in Fourier-transform infrared spectroscopy (FT-IR) (Fig. 2a). The FT-IR of pure regenerated SF (RSF) was determined to correlate the changes, which occurred in the vibration modes of the Sil-MA. The spectra in both RSF and all Sil-MA samples showed peaks of amide I, II, and III at 1639, 1512, and 1234 cm^−1^, respectively. A weak band in Sil-MA groups was visible at 1238 cm^−1^, which indicates CHOH stretching of the alcohol group, which was produced after the opening of the epoxy group of the GMA. The other small changes detected in the Sil-MA specimens were at 951 and 1165 cm^−1^, representing CH_2_ wagging stretching of methacrylate vinyl group in the GMA. These peaks gradually increased with greater quantities of GMA. The GMA functional groups were only slightly detectable or hidden by SF peaks because the molecular weight of GMA is much smaller than that of the SF.

The degree of methacrylation was evaluated and the resulting, abovementioned ring-opening of epoxy at GMA and nucleophilic addition reactions by primary amine on lysine depending on GMA amounts was proved by proton nuclear magnetic resonance (^1^H-NMR) spectroscopy (Fig. 2b). Specifically, we found that the characteristic resonance of the methacrylate vinyl group (*δ* = 6.2–6 and 5.8–5.6 ppm) and the methyl group of GMA at *δ* = 1.8 ppm has appeared by addition of the GMA, and the integration of these peaks increased with the addition of greater GMA amounts. In addition, there was a gradual decrease of the lysine methylene signal at *δ* = 2.9 ppm with increasing quantities of GMA, which indicated modification of the lysine residues in SF (22–42%; Table 2). Lastly, measurable signals were found at *δ* = 3.2–3.6 ppm resulting from the hydrogen neighboring the C–N bond.

**Table 2:** Major peak integration in silk fibroin (SF) modified by glycidyl methacrylate (GMA) (Sil-MA) and degree of methacrylation on SF

Groups	Methacrylate vinyl group	Methyl group	Lysine	Methacrylation degree (%)
RSF	0.1	0.26	1.43	0.0
Sil-MA-141 mM	0.23	0.55	1.11	22.4
Sil-MA-282 mM	0.35	0.59	0.97	32.2
Sil-MA-424 mM	0.75	0.87	0.83	42.0
Sil-MA-705 mM	0.51	0.77	0.87	39.2

GMA was added at a concentration of 141, 282, 424, and 705 mM to SF

The microstructures of the Sil-MA hydrogels were observed by scanning electron microscope (SEM; Fig. 2c). The SEM images revealed uniform and interconnected porous microstructures throughout all the samples. The pore size showed decreasing pattern with higher degrees of methacrylation and Sil-MA concentration (Supplementary Fig. 1). It has been previously reported that the degree of methacrylation affects the physical and mechanical properties of hydrogels, with higher methacrylation resulting in stiffer and tougher hydrogels and smaller pore sizes^31^. Based on these results, a schematic representation illustrating the network formation was created (Fig. 2d).

### Mechanical properties and water uptake of Sil-MA

**Fig. 3:**
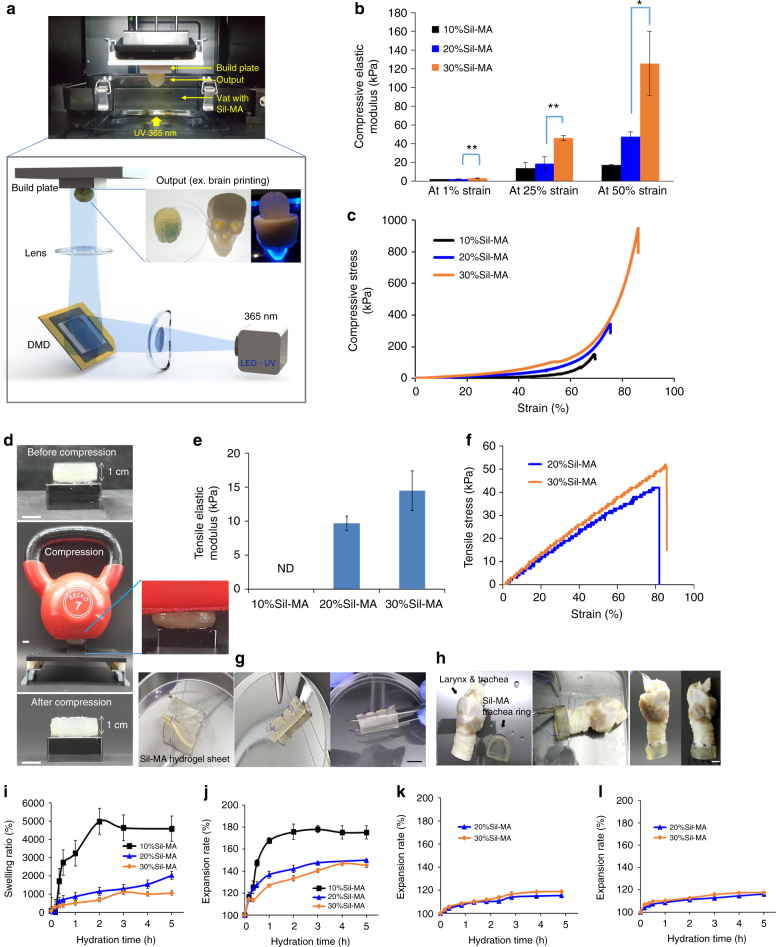
Physical properties of Sil-MA hydrogel. **a** Schematic diagram of DLP bioprinting procedure using Sil-MA. Sil-MA-added LAP was printed in a layer-by-layer style with DLP printer. Herein, the desired pattern was designed through CAD software and was sliced to a layer file before transferring it to the DLP system. DMD digital micromirror device. **b**–**d** Compression test for Sil-MA hydrogel with varied Sil-MA contents. **b** Compressive elastic modulus of Sil-MA hydrogel at different percentage strains. Significant differences are presented. *p < 0.05 and **p < 0.005 (two-sample *t*-test). **c** Representative compressive stress–strain curve and **d** the Sil-MA hydrogel was compressed by a kettle bell (7 kg) for 3 min and recovered hydrogel’s initial shape after it was removed. **e**–**h** Tensile stress for Sil-MA hydrogel. **e** Tensile elastic modulus of Sil-MA hydrogel at 50% strain and **f** representative tensile stress–strain curve of Sil-MA hydrogel. **g** Suturing of folded lumen structure of 3D-printed Sil-MA membrane and **h** trachea end-to-end anastomosis of dog’s larynx and trachea using Sil-MA 3D DLP-printed hydrogel. Scale bar indicates 1 cm. **i**–**l** Swelling properties. **i** Water uptake of Sil-MA powder. Volume expansion rate of DLP product in **j** water, **k** medium, and **l** PBS (pH 7.4). Data are presented as mean ± s.d. The black line (/bar), blue line (/bar), and orange line (/bar) indicate 10%, 20%, and 30% of Sil-MA hydrogel, respectively. Each assay was conducted in triplicate

Sil-MA hydrogel for mechanical testing was fabricated by the DLP-manufacturing machine using a Sil-MA slurry, including lithium phenyl(2,4,6-trimethylbenzoyl) phosphinate (LAP; Fig. 3a). A UV light intensity of 3.5 mW cm^−2^ for 3 s was sufficient to solidify the solution in each layer. We confirmed the mechanical suitability of Sil-MA hydrogel by obtaining stress–strain curves for compression and elongation (Supplementary Fig. 2a-b). The compressive elastic modulus increased with increasing Sil-MA concentration and strain (Fig. 3b). Every 10% increment of Sil-MA concentration brought about 2.6-fold increases in the compressive stress (Fig. 3c). The compressive strain and the compressive stress at break increased according to the Sil-MA concentration. A particularly impressive outcome (910 kPa at break) was observed for the 30%Sil-MA. The 30%Sil-MA hydrogels were strong enough to support the weight of a kettle bell (7 kg) and returned to their original shape immediately after the kettle bell was removed (Fig. 3d). The higher concentration of Sil-MA hydrogels resulted in increased tensile strength and breaking elongation. Sil-MA’s tensile elastic modulus was also increased with greater Sil-MA concentrations (Fig. 3e). The tensile strength of the 30%Sil-MA was 1.5 times higher, and the breaking elongation measured 1.6 times more than that of the 20%Sil-MA (Fig. 3f). We were able to suture using 30%Sil-MA. The 3D-printed Sil-MA membrane was folded slightly and then sutured using simple interrupted suture without tearing (Fig. 3g and Supplementary Movie 1). Furthermore, we were able to complete successfully an end-to-end anastomosis using 3D-printed 30%Sil-MA tracheal rings with subsequent continuity (Fig. 3h and Supplementary Movie 2).

In the water uptake test, specimen weight was increased with lengthening of the hydration time up to 5 h, however, after that a constant weight was obtained for all Sil-MA hydrogels. It was calculated that the Sil-MA hydrogel had high water uptake 46 times greater with 10%Sil-MA hydrogel, 20 times greater with 20%Sil-MA hydrogel, and 11 times greater with 30%Sil-MA hydrogel compared to each Sil-MA hydrogels’ dry weight (Fig. 3i and Table 3). The volume expansion measurements in water (Fig. 3j and Table 3) also showed an increase in size of 150 ± 0.9% for 20%Sil-MA hydrogel and 145.4 ± 2.4% for 30%Sil-MA hydrogel, both of which were lower than that of 10%Sil-MA hydrogel (175.2 ± 6.1%). On the other hand, in medium without supplemental ingredient and in phosphate-buffered saline (PBS; pH 7.4), their volume was not expanded as much as in water (Fig 3k, l).

**Table 3:** Mechanical properties of Sil-MA hydrogel depending on Sil-MA contents

Sil-MA contents (%)	10	20	30
Compressive stress at break (kPa)	122 ± 45	434 ± 128	910 ± 127
Compressive strain at break (%)	69.5 ± 0.5	77.7 ± 3.3	80 ± 5.1
Compressive elastic modulus at 50% strain (kPa)	17.7 ± 0.3	47.8 ± 4.8	125.8 ± 34
Tensile stress at break (kPa)	ND	52 ± 4.3	75 ± 7.5
Elongation at break (%)	ND	77.6 ± 3.8	124.2 ± 41
Tensile elastic modulus at 50% strain (kPa)	ND	9.7 ± 1.0	14.5 ± 2.9
Water content at 5 h from dried Sil-MA (%)	4580 ± 480	2026 ± 202	1059 ± 121
Expansion rate at 5 h (%)	175.2 ± 6.1	150.0 ± 0.9	145.4 ± 2.4

### Rheological properties of Sil-MA

**Fig. 4:**
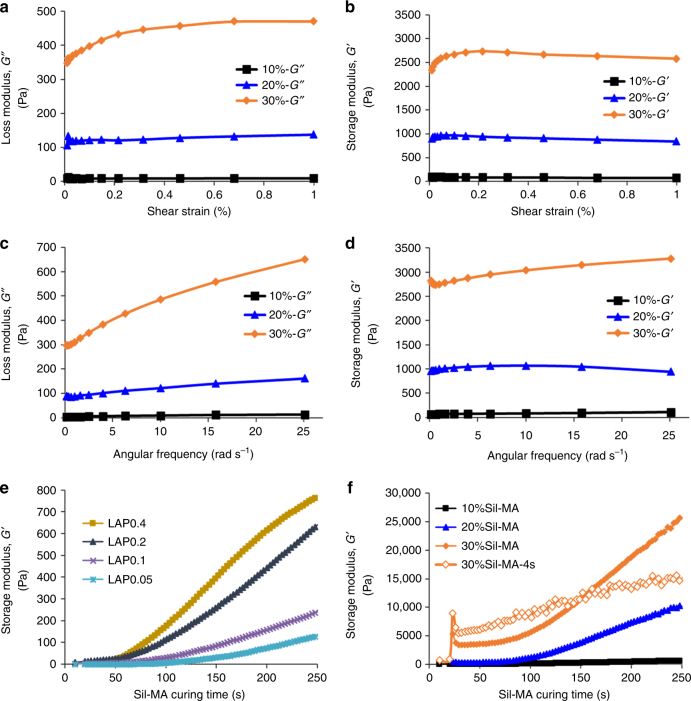
Rheological analysis for Sil-MA hydrogel. **a**, **b** Strain dependency and **c**, **d** frequency dependency of **a**, **c** loss modulus (*G*″) and **b**, **d** storage modulus *G*′ for Sil-MA hydrogels at different Sil-MA contents varied from 10 to 30%. **e**, **f** In situ rheology during UV exposure. The effect of **e** LAP contents and **f** Sil-MA contents on the *G*′ during UV exposure for 250 s except for the line with orange squares (30%Sil-MA) exposed for only 4 s. The black line, blue line, and orange line indicate 10%, 20%, and 30% of Sil-MA hydrogel, respectively

To characterize the rheological properties of the Sil-MA hydrogels, an oscillatory rheological measurement was used. This test was carried out by varying the amplitude or frequency in a series of trials. For the amplitude sweep (Fig. 4a, b), the condition was set at 0.01–1% strain and a constant angular frequency (10 Hz). Storage modulus (*G*′) and loss modulus (*G*″) represent the elastic part and the viscous part for Sil-MA hydrogels, respectively. Up to 1% shear strain, most hydrogels showed nearly constant values for *G*′ and *G*″, which suggests that the structures in all specimens were undisturbed. The peak gradually rose with increasing Sil-MA concentration and the most marked peak was shown in the 30%Sil-MA hydrogel. In the all groups, *G*′ was larger than *G*″ by a factor of approximately 6–8 in the linear viscoelastic ranges regardless of the Sil-MA concentration, which meant that the Sil-MA gels behaved as elastomeric materials. Of note, the Sil-MA hydrogel closely resembled a solid given the phase angle (*δ*) of 6.4–9.1° (Supplementary Table 1). The *G*′ and *G*″ at 0.158–25.1 Hz of the frequency range and 1% deformations were measured. The properties of the material demonstrated minimal changes in stress at low frequencies, and greater changes at high frequencies. The increasing pattern of *G*″, which indicates weakly crosslinked Sil-MA in the network, was shown to a minimal degree during oscillation (Fig. 4c). The highest *G*′ among Sil-MA groups was about 3 kPa in the 30%Sil-MA hydrogel (Fig. 4d).

The rheological properties of Sil-MA were monitored during the UV-curing process to assess the kinetics of the photocrosslinking reaction at 0.1% strain and 1 Hz frequency. They were measured both for the combination of varying photo-initiating concentration with constant Sil-MA content and for varying Sil-MA concentration under constant photo-initiating concentration. Figure 4e shows that Sil-MA hydrogels tended to become stiffer with increasing curing times, which meant that longer exposure to UV light resulted in greater stabilization of the hydrophobic domains when SF was methacrylated by GMA. The *G*′ at 250 s corresponding to 3.5 mJ cm^−2^ of a UV energy density reached 130, 241, 642, and 771 Pa in the condition with LAP 0.05%, 0.1%, 0.2%, and 0.4%, respectively, and increased up to 642, 10 151, and 25 577 Pa as the Sil-MA concentration was increased from 10% to 20% and to 30%, respectively (Fig. 4f). Meanwhile, when UV was turned off after 4 s treatment in the 30%Sil-MA, the increase rate of *G*′ was low. Supplementary Table 2 shows gel point depending on LAP or Sil-MA concentration. The starting point of gelation decreased from 101 to 41 s with increasing LAP concentration and rose from 74 to 137.5 s with increasing Sil-MA concentration.

### The actual resolution and printability of Sil-MA via DLP printing

To get the actual resolution and accuracy of 3D DLP printing using Sil-MA, we measured the actual figure size after printing and determined the deviation by comparing the nominal diameters in both the horizontal and vertical planes. The observable features were formed when nominal *X* and *Z* dimensions were at least 100 and 300 µm, respectively (Supplementary Fig. 3a). Our system produced spatial accuracy with an average deviation of 66 µm (circle diameter), 90 µm (square width), and 142 µm (height) (Supplementary Fig. 3b-d).

**Fig. 5:**
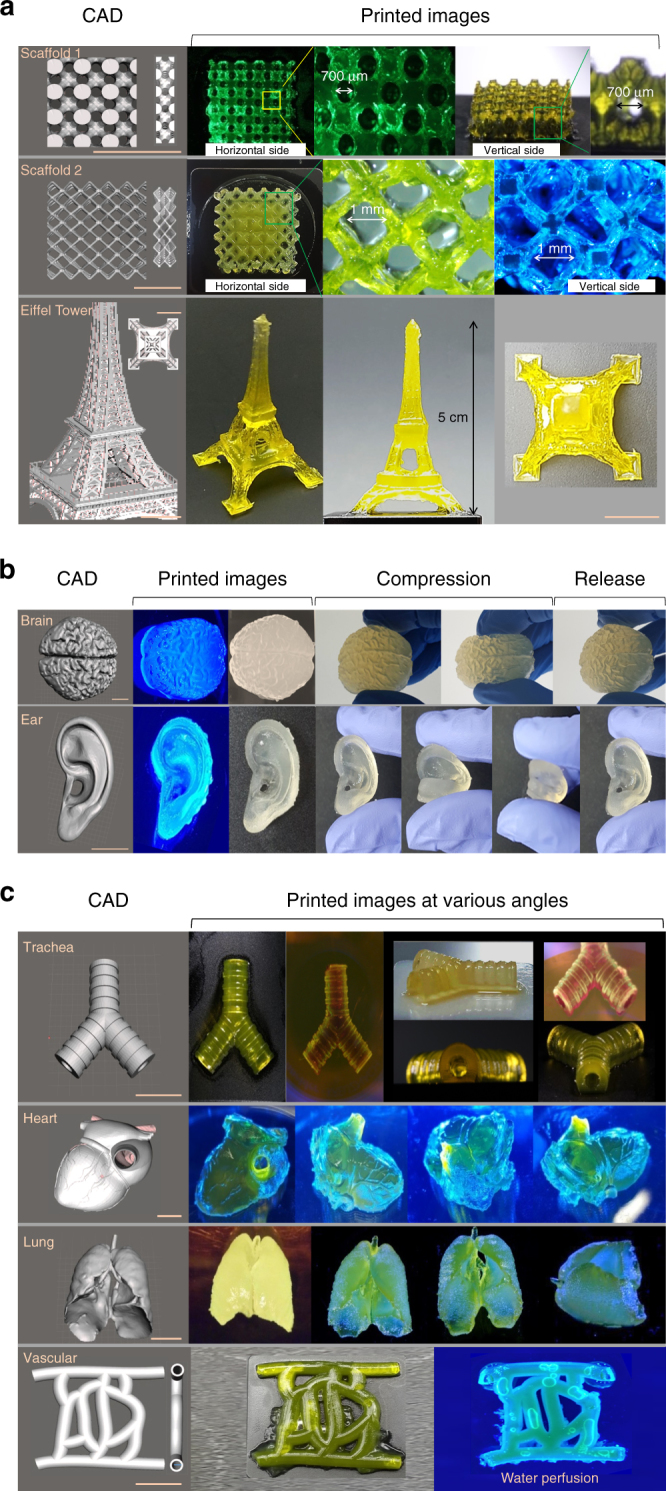
Printability of 30%Sil-MA using DLP printer. **a** Porous scaffold and Eiffel Tower imitation; (l) CAD images depicting scaffolds and Eiffel Tower and (r) printed images. Printed scaffolds had small pores around ~700 μm and Eiffel Tower had small holes and grid on the surface. **b** Ear and brain mimicked shape; (l) CAD images depicting the ear and brain and (r) printed images. Printed products were not damaged when they were compressed by fingers tightly and they were back to their original shape when relaxed his fingers. **c** Trachea, heart, lung, and vessel mimicked shape; (l) CAD images depicting the trachea, heart, lung, and vessel and (r) printed images at various angles. Printed products by DLP using Sil-MA showed complex structure reflecting their CAD images, including veins, arteries, folds, and holes. Scale bar indicates 1 cm

We chose the 30%Sil-MA for the printability test because it printed as designed for both pattern and size, as well as ease of handling after DLP printing (Supplementary Fig. 4). We mimicked various complex and difficult shapes to print as examples of the versatility of 30%Sil-MA using DLP printer. All printed structures mirrored the forms of the designed CAD images. Mesh style scaffolds with small (~700 μm) and highly interconnected pores visible to the naked eye were printed (Fig. 5a). In addition, a replica of the Eiffel Tower in miniature was accurately constructed by the DLP printer. Figure 5b, c shows that DLP printing using 30%Sil-MA would be capable of constructing complex organ structures: ear auricle with helical fold; the cerebral sulcus and grooves; the lumen inside the trachea and joints on it; the heart with aorta and pulmonary artery/vein; and the lung with thoracic cavity and the vascular network structures with small caliber. In addition, when the printed products, including the brain and ear were distorted by pressure from the thumb and index finger, they returned to their original shape without residual deformity (Supplementary Movies 3 and 4). Finally, patency of the 1.6 mm-sized blood vessels in the different models was demonstrated through perfusion.

### Cytocompatability of Sil-MA hydrogel

**Fig. 6:**
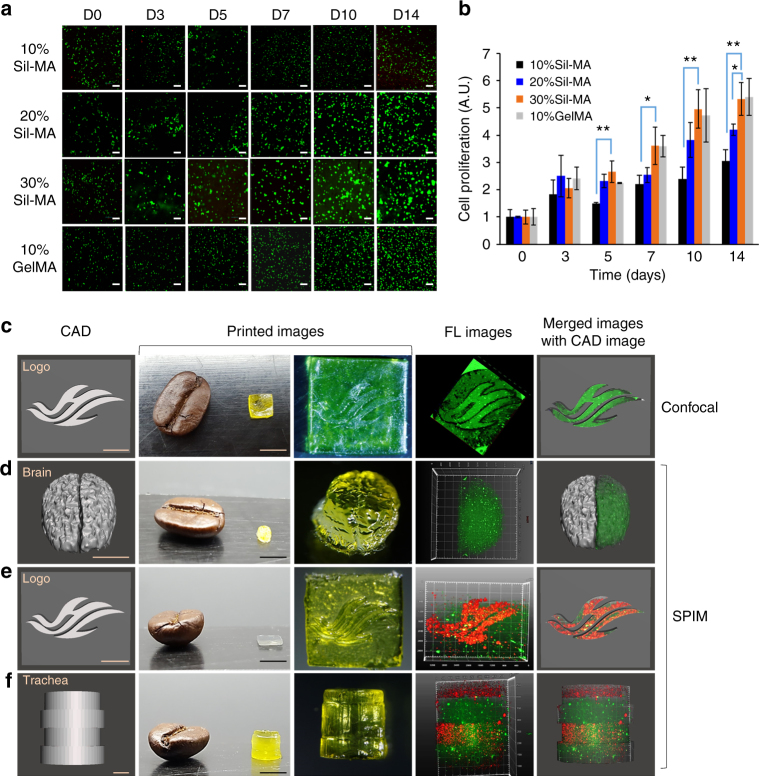
Cytocompatibility of Sil-MA. **a** Live and dead assay; cell viability of encapsulated NIH/3T3 for 14 days (live cells in green and dead cells in red). Scale bar indicates 500 μm. **b** CCK-8 assay; cell proliferation in the hydrogel for 14 days. The 30%Sil-MA hydrogel showed the similar cell proliferation to that of commercial GelMA (10%). * and ** refer to statistically significant proliferation *p* < 0.05 and *p* < 0.005 (two-sample *t*-test), respectively, compared to 30%Sil-MA hydrogels. The black line, blue line, orange line, and gray line indicate 10%, 20%, and 30% of Sil-MA hydrogel and 10%GelMA hydrogel, respectively. **c**–**f** Cell distribution inside the Sil-MA hydrogel. Cells were distributed evenly in the hydrogels. The Sil-MA hydrogels with **c** a design of the letter HL (the logo of Hallym University) and **d** a shape of human brain were printed out with PKH67-labeled cells only. Also, the Sil-MA hydrogels with **e** the letter HL and **f** a shape of winding trachea were printed out with PKH67-labeled cells (green) and PKH26-labeled cells (red); (from left to right) CAD images, printed images, fluorescence images by **c** confocal or **d**–**f** single plane illumination microscopy (SPIM) microscope, and merged images of fluorescence and CAD images. Scale bar indicates 1 mm on CAD images and 5 mm on printed images. Data are presented as mean ± s.d. Each assay was conducted in triplicate

Live/dead assay was carried out to evaluate the cytocompatibility of the Sil-MA hydrogel using NIH/3T3 fibroblast for potential cell encapsulation applications (Fig. 6a). Cells were suspended in Sil-MA solution (10 ~ 30%) or commercial GelMA solution (as a control group, 10%). This cell suspension was used for printing by a DLP printer and the printed ones were cultivated for 14 days. Cells encapsulated inside Sil-MA hydrogels remained mostly viable regardless its concentration, and a mild aggregation was detected with increasing concentration of Sil-MA due to strong interaction between cells by decreasing pore sizes (Fig. 2c). The average pore sizes are inversely related to the concentration of hydrogel materials^30^. CCK-8 assay demonstrated that the cells grew well in the Sil-MA hydrogel and that the cell proliferation was as high in the 10%GelMA as in 30%Sil-MA (Fig. 6b).

Figure 6c–f demonstrates cell distribution into the Sil-MA hydrogel through DLP printing. It was found that cells were distributed evenly over the printed constructs regardless their size and complexity of structure from four specimens. In the logo printing, the embossed logo (thickness 400 μm) against a bottom sheet was printed out clearly with even cell distribution (Supplementary Movie 5). Second, in brain construction, the cells labeled with green fluorescence were printed without obliteration of the small grooves and sulcus on it (Supplementary Movie 6). Third, in the logo printing model using cells labeled with two colors, cell position could be controlled as designed (Supplementary Movie 7). Rather than simply printing a one- to two-layer structure, we printed a colorful trachea of four layers with different colors on each layer with good integrity and even cell distribution (Supplementary Movie 8). Most notable is that cell distribution and clear printing as designed were accomplished although all the printing size was close to a tenth of a coffee bean.

### Cartilage tissue engineering using Sil-MA hydrogel

**Fig. 7:**
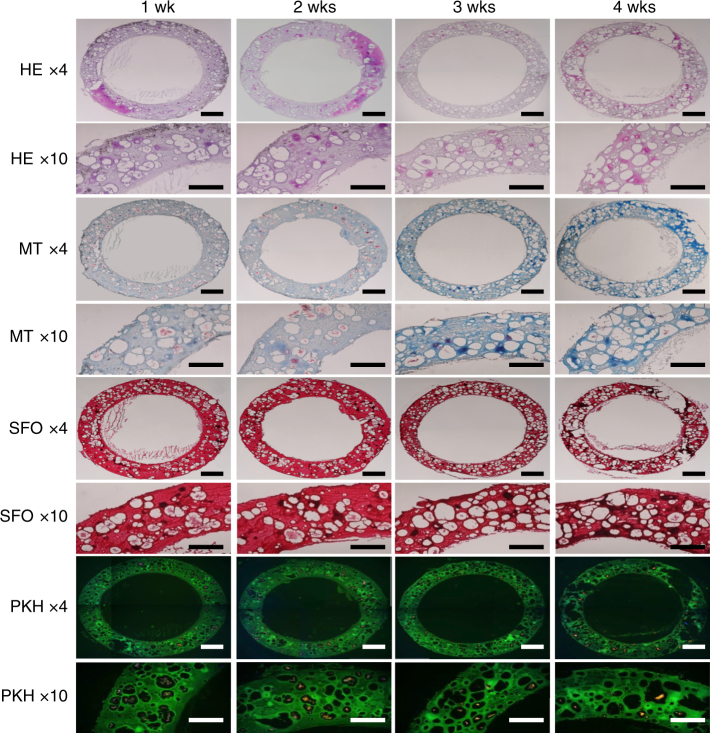
In vitro histological evaluation of human chondrocytes loaded Sil-MA hydrogel for cartilage tissue engineering. Scale bars represent 1 mm (×4) and 500 μm (×10). Sil-MA hydrogel showed superior histological characteristics (cell organization and extracellular matrix distribution, including proteoglycan and collagen) of cartilage-like tissue with time in vitro. HE H&E, MT Masson’s trichrome, SFO Safranin-O, PKH PKH26 (red: chondrocytes, green Sil-MA hydrogel)

For long-term biocompatibility of Sil-MA, we fabricated a ring-like cartilaginous trachea by DLP printer and tested it in vitro. Figure 7 shows in vitro result at each time point of 1, 2, 3, and 4 weeks to demonstrate in vitro cartilage tissue formation. Cells were distributed evenly over the Sil-MA hydrogel and exhibited rounded morphology with lacunae embedded in basophilic extracellular matrix when the cell-laden Sil-MA hydrogel was stained using Hematoxylin and Eosin (HE). The pericellular region and interterritorial matrix region were densely stained by the typical red of Safranin-O indicating proteoglycan-rich matrix and by blue distinctive of Masson's Trichrome (MT) indicating the collagen matrix. Closely packed chondrocytes with lacunae were notably observed and the matrix around them was progressively stained over time. In the fluorescence microscopy examinations, the visible red signal demonstrated the presence of chondrocytes for up to 4 weeks in vitro, which was consistent with the histological data. We evaluated degradation rate of 30%Sil-MA hydrogel with its potential value for DLP printing. Supplementary Fig. 5 shows degradability of 30%Sil-MA hydrogel with cells in vitro. Cell-loaded Sil-MA hydrogel degraded gradually and exhibited a 50% degradation rate at 4 weeks after cultivation. These results indicate that the Sil-MA hydrogel provided an excellent environment for the growth of chondrocytes and cartilage formation in vitro.

## Discussion

DLP additive manufacturing has the potential to contribute to improved fabrication of complex structures in tissue engineering. However, DLP printing for tissue engineering would not be possible in the absence of photocrosslinkable bioinks possessing good mechanical strength and biocompatibility.

SF produced by *B. mori* is one of the strongest fibrous proteins in nature with biocompatibility and biodegradable properties. With that said, most of the silk materials developed from RSF solution have ordinary mechanical properties compared to native silk fibers due to the degumming and dissolution processes. Therefore, the RSF has been enhanced mechanically through various processing strategies, including increase of temperature, methanol/salt treatment, physically blended or chemically crosslinked with other polymers, and so on. Several studies have been tried to fabricate a photocrosslinked hydrogel based on SF by combining another materials introduced methacrylated groups (e.g., GelMA and poly(vinyl alcohol) methacrylate)^32,33^. However, there have not yet been studies of photocuring sites being introduced directly into the SF itself. Though one study about photocrosslinkable SF, which uses riboflavin as a photoinitiator and visible light as a light source^34^ has been reported, the visible light, which is needed for longer treatment (minutes), and has a high penetration ratio is not suitable for the DLP printer to create the detailed patterns required. Therefore, a more suitable modification method for SF is necessary for the use of the DLP technique.

Previously, we modified the timing of adding GMA to the SF. From this we found that the early addition of GMA into the SF solution produced the best Sil-MA through an unknown mechanism. This is different from the usual methacrylated gelatin (GelMA)-manufacturing method. We also found that final quality of Sil-MA depends on the reagent used for methacrylation. We applied both MA and GMA, reagents commonly used in methacrylation of gelatin, to SF. However, the crystallization of the SF during either the reaction or dialysis was observed when MA was applied to the SF. We speculated that one possible reason for this phenomenon is that a reaction by-product, methacrylic acid, reduces the pH of the SF solution during the reaction. As a result, the free amino groups of SF could become ionized, which hinders the reaction with MA^35^. With regards to the pH reduction, the protonation of the side chain carboxyl group that plays a principal role in determining gelation rate could be expected. This protonation would neutralize the carboxyl groups, reduce hydrophilicity, and decrease charge repulsion, which accelerate the hydrophobic interactions of the fibroin molecules and resultingly produce increased physical crosslinks and faster gelation^36^. In contrast, application of GMA resulted in a stable reaction. We believe that the reaction using GMA was stable without acidic by-product through the mechanism of epoxide ring-opening (as a major mechanism)^37^. In addition, GMA could be to increase the reactions with the amino groups. Theoretically, two GMA molecules can react with one free amino group (Fig. 1a), while only one MA molecule reacts with one free amino group of the lysine residue^38^. Therefore, the use of GMA maximizes the number of vinyl groups on SF which contains only small quantities of lysine (0.2 mol%)^28,39^. GMA can also react with the hydroxyl and carboxyl groups in SF molecules. These functional groups can facilitate further methacrylation of SF through the transesterification mechanism (as a minor mechanism)^36^, increasing the amount of vinyl methacrylate, and thus maximizing the crosslinking density of Sil-MA hydrogels^40^.

We varied the amount of GMA added to the SF solution to find the suitable methacrylation degree with minimal ingredients. SF (~350 kDa) has 0.2 mol% lysine, which is likely the main site for the introduction of the GMA into SF^28,39^_._ In this reaction, SF would be coupled to the pendant active epoxide groups of the GMA by nucleophilic reaction between –NH_2_ moieties in the lysine on α-helix or β-sheet of SF and epoxide groups^41,42^. Testing of the Sil-MA through FT-IR and ^1^H-NMR revealed that GMA has been successfully grafted to the SF molecules (Fig. 2a, b). Overall, an increase in GMA concentration resulted in higher level of methacrylation of SF, which could be seen by the decrease in the lysine methylene group (Table 2). The degree of methacrylation affects the physical and mechanical properties of hydrogel such as stiffness, pore size, and durability of hydrogel^31^. Sil-MA hydrogel’s mechanical properties can be tuned by adjusting GMA concentration depending on target tissue. However, there was no significant effect of increased amounts of GMA beyond 424 mM. We suggest that the ideal GMA concentration is from 282 to 424 mM and it can be modified depending on applications, based on the required mechanical properties. In this study, 424 mM of GMA showed maximum methacrylation rate and was selected for the various mechanical and cell viability studies. We assumed that unreacted lysine was shielded by the reacted sites as the reaction progressed and that the GMA would then react with other amino acids beside lysine. In fact, striking signals at *δ* = 3.2–3.6 ppm assigned to hydrogen neighboring C–N bond were found. This result indicates that reactions occurred between the secondary amines in the amino acids of SF and GMA even though their reactivity is not as strong as in the primary amine. It has been reported that SF contains 1105 of the 5000 reactive amino acids, including lysine 0.2%, arginine 0.3%, asparagine 0.4%, and glutamine 0.2% in its primary amino-acid group and histidine 0.1% and tryptophan 0.2% in the secondary group^28,39^.

The fabricated Sil-MA allowed fabrication of the 3D structures using a DLP printer (Fig. 3a). A photoinitiator, LAP, was added to photopolymerize Sil-MA. LAP has greater water solubility and low cell cytotoxicity^43^. The photopolymerization reaction was initiated by LAP mixed with a Sil-MA solution and exposed to UV light of 365 nm. LAP produces a free radical that attacks vinyl monomers of Sil-MA, which serve as a key linkage. It leads to radical-initiated chain polymerization since the surplus electrons will occur on the either end of the vinyl bond^44,45^.

Mechanical properties of the tissue-engineered product influences the cellular interactions and cell functions^46^. The capacity of 3D-printed scaffolds to resist compressive loads is important given the frequent forces on the various structures and organs of the body. In this study, increasing the Sil-MA concentration resulted in enhancement of mechanical characteristics. Here, 30%Sil-MA showed the strongest mechanical strength and excellent resiliency following distortion among groups (Fig. 3b–d). The compressive strength of 30%Sil-MA hydrogel was about 10 times higher compared to PCL blended gelatin hydrogel as a synthetic/natural hybrid scaffold (75–94 kPa)^47^. The compressive elastic modulus of 30%Sil-MA was about 1.5 times higher than that of 30%GelMA (88 kPa; Supplementary Fig. 6). Based on its elastic modulus, pure Sil-MA hydrogel could be applied to the creation of 3D-printed artificial mitral valve leaflets (<100 kPa)^48^, smooth muscle (6–10 kPa), and carotid artery (84 kPa). Sil-MA hydrogel also produced structures with highly flexible and stretchable properties as the concentration of Sil-MA was increased (Fig. 3e–h). Therefore, Sil-MA could be applied to a variety of clinical applications based on its acceptable mechanical properties.

We expect that the improved mechanical properties could be associated with physical entanglement of SF molecules, in addition to the chemical methacrylation of the SF. First, as Sil-MA content increases, the higher concentration of Sil-MA macromolecules in the Sil-MA solution raises the chances of chemical linkage and entanglements among the macromolecules. Second, the stimuli from this physicochemical entanglement between Sil-MA molecules could promote secondary crystalline conformation (β-sheet) more likely, generating added strength and new hydrophobic regions within the SF^49,50^. Based on these assumptions, possible linkages affecting the strength and resilience of the Sil-MA hydrogel were drawn as a 3D structure in Fig. 2d and are described as follows: (1) covalent intra-chain linkages; (2) chain–chain linkages through introduced vinyl double bonds; and (3) physical entanglement between the multiple long chains of SF macromolecules in the hydrogel.

Water uptake ability of hydrogel is important from the point of view of the affinity of the manufactured materials on the adjacent tissues. At the same time, the retention of shape is also necessary for medical applications. Sil-MA hydrogel had excellent swelling properties, but they were decreased with increasing Sil-MA contents (Fig. 3i–l). This limitation of water uptake and volume expansion with increasing Sil-MA concentration could be associated with the increased mechanical strength mentioned above.

Rheological properties of DLP-printed Sil-MA hydrogel, such as loss and storage modulus and the correlation between Sil-MA concentration and photoinitiator catalyst in terms of the starting point for gelation were confirmed controlling for LAP and Sil-MA content (Fig. 4). Data showed that the Sil-MA hydrogel was close to solids through the phase angle (Supplementary Table 1). Commonly, the phase angle is related to the viscoelastic character of materials. The phase angle is zero for a purely elastic material (solid-like behavior) and 90° for a purely viscous material (liquid-like behavior). This characteristic is important for the medical requirements that must be fulfilled when using Sil-MA as a tissue replacement substance^51^. It was noted that increases in LAP augmented Sil-MA content and advanced the gelation time; however, this approach should be limited to avoid possible cytotoxicity.

In the laboratory, organs and tissues with tubular (e.g., trachea and vascular networks) as well as solid organs (e.g., ear, brain, heart, and lung) were mimicked with good mechanical integrity using 30%Sil-MA in a 3D DLP printer (Fig. 5). Current bioprinting techniques have technical challenges in regards to facilitating vascularization, innervation, and viable cell distribution within complex 3D tissues. All of these characteristics are essential for biological and clinical application of 3D printing to tissue engineering. Most researchers today, in order to create the lumen of the vasculature network, use extrusion printing with a sacrificial ink that creates the 3D spaces and then is resorbed after the outline is complete^52^. Alternatively, a coaxial nozzle can be used to make hollow fibers, but branched structures cannot be produced using this method^53^. In contrast, a DLP printing system with Sil-MA enables complex tubular structures with multiple branches (including capillary networks on the surface of the heart) to be created in one procedure without a complicated second set of steps. Our system facilitated structures that were not only stable, but layered up to 45–50 mm (e.g., brain and Eiffel Tower) significantly higher than what is possible with alternative hydrogels. This major advance in 3D printing results from the harmony between the unique properties of Sil-MA and the layering capability of DLP technology. The maximum height capable of being achieved with other hydrogels is limited to 3–10 mm using FDM and DLP modalities^54–56^.

DLP 3D-printed Sil-MA hydrogel could be a promising biomaterial in tissue engineering due to its excellent biocompatibility. Cells were not damaged because of rapid printing speed (3 s per layer) with accuracy, as a result, the cells grew well and were distributed equally over the hydrogel set, an outcome important for biological and clinical applications (Fig. 6). Furthermore, we demonstrated multilayer printing using different cells, replicating biological tissue, which consists of multi cell types. Appropriate viscosity is also important during 3D printing. DLP printing requires relatively low viscosity during the printing itself compared to extrusion techniques, but tissue engineering also needs higher viscosity to avoid deformation and collapse of the final product. Thirty percent Sil-MA with UV crosslinking fulfilled both of these requirements with excellent cell growth and detailed final structures. Also, the cell concentration of 1–2 × 10^7^ cells per mL did not affect the integrity of the final construct. From the in vitro study using human chondrocyte, we were able to observe degradation of the 30%Sil-MA hydrogel up to 50% for 4 weeks and the positive environment for chondrocytes in the hydrogel (Fig. 7).

It is worthy of note that Sil-MA bioink uses only naturally occurring polymers, rather than most of the alternatives, which are a combination of a natural polymer with a synthetic material^57,58^, and yet the Sil-MA hydrogel produces equivalent strength and stability. This study suggests that photopolymerizable Sil-MA would be a superb bioink for DLP printing for biological and clinical applications.

## Methods

### Preparation of methacrylated SF powder and hydrogel prepolymer

*B. mori* cocoons were obtained from the Rural Development Administration (Jeonju, Korea). Each silkworm cocoon was sliced into four pieces. A unit of 40 g of sliced cocoons were boiled in 1 L of 0.05 M Na_2_CO_3_ solution for 30 min at 100 °C to remove the sericins, and then washed with distilled water several times. Figure 1b is a schematic diagram illustrating the preparation and photopolymerization of the Sil-MA used for the DLP printer. Subsequently, degummed silk was dried at room temperature and 20 g of it was dissolved in 100 mL of 9.3 M lithium bromide (LiBr) solution at 60 °C for 1 h. Right after SF was solved by LiBr, 2, 4, 6, and 10 mL (141, 282, 424, and 705 mM) of GMA solution (Sigma-Aldrich, St. Louis, USA) were added to the mixture stirring with a speed of 300 rpm for 3 h at 60 °C to create a high yield reaction between GMA and SF. Then, the resulting solution was filtered through a miracloth (Calbiochem, San Diego, USA) and dialyzed against distilled water using 12–14 kDa cutoff dialysis tubes for 4 days. Finally, methacrylated SF solutions were frozen in −80 °C for 12 h and freeze-dried for 48 h. Lyophilized methacrylated SF (Sil-MA) powder was stored −80 °C for further use.

### Characterization (NMR/FT-IR/SEM)

To determine the degree of methacrylation, the Sil-MA and varying amounts of GMA were examined through ^1^H-NMR at a frequency of 400 MHz using a Bruker DPX FT-NMR Spectrometer (9.4 T) of Bruker Analytik GmbH company (Karlsruhe, Germany) and 700 μL deuterium oxide (D_2_O, Sigma-Aldrich) as the solvent per 5 mg sample^59.^ Sil-MA solution was filtered using a 0.45 μm filter before analysis. ^1^H-NMR spectra were recorded for unsubstituted SF (RSF) and GMA with 1:0.7, 1:1.4, 1:1, and 1:3.4 mol unsubstituted amines on silk:mol GMA. Baseline correction was applied before measuring the integrals of the peaks of interest with ACD/NMR Processor Academic Edition. The degree of methacrylation was defined according to the percentage of ε-amino groups of lysine in SF that are modified in Sil-MA. For this, the signal from the protons produced by the aromatic amino acids in Sil-MA at 6.9–7.5 ppm was used to normalize each spectrum. Then, the lysine methylene signals (2.8–2.95 ppm) of samples were integrated to obtain the areas. The degree of methacrylate substitution was determined by the formula: 1 − (lysine integration signal of Sil-MA/lysine integration signal of unsubstituted SF) × 100^29^. FT-IR spectra can be a valuable tool in studying graft copolymerization reactions. The structure of Sil-MA was confirmed by FT-IR spectra (Frontier, PerkinElmer, Rodgau, UK). Samples were prepared by grinding the samples with potassium bromide (KBr). The microstructures of the Sil-MA hydrogels were observed under a field-emission SEM (VP-FE-SEM; EVO^®^LS10, Carl Zeiss, Germany) at the Korean Basic Science Institute (KBSI, Chuncheon, Korea). Printed Sil-MA hydrogel by 3D DLP printer was freeze-dried. The samples were coated with a thin 10 nm layer of gold/palladium for 30 s at 15 mA discharge current (Ion Sputter 1010, Hitachi, Japan). The micrographs were obtained at an accelerating voltage of 1.2–1.3 kV.

### DLP 3D printing

Our photocurable hydrogel formulation was used in a DLP projector. Freeze-dried Sil-MA (prepared by 424 mM of GMA) was dissolved in water at various concentrations of 10–30% w/v and the photoinitiator was added and mixed until fully dissolved. In these experiments, the photoinitiator, LAP (Tokyo chemical industry, Tokyo, Japan) was used at a concentration of 0.2% w/v in hydrogel prepolymer (Fig. 1b).

Over-curing of layers beyond the focal plane should be prevented to produce the best-quality 3D structures. To minimize this problem, a high-quality DLP printer of laboratory scale was designed and fabricated (Fig. 3a). Our DLP printing system consists of three major components: UV Digital Micro-mirror Device™ with a resolution of 30 µm (1920 × 1068 × 1080 pixels; Texas Instruments, Dallas, USA), 365 nm UV-LED (LG Innotec, Seoul Korea) with average 30 mW cm^−2^ intensity, and a lens module (focal length 60 mm, aperture f/2.8, distortion < 80%, offset 0%, working distance 130 mm, and field of view 28.8 × 16.5 mm) with two UV-grade biconvex lenses (24 mm diameter). The build area is 35 (*L*) × 20 (*W*) × 120 (*H*) mm with a layer thickness adjustable from 5 to 200 µm. The system was customized by professional manufacturers (NBRTech. Ltd, Chuncheon, Korea and Illuminaid. Ltd, , Seongnam, Korea).

Specimens were designed by CADian3D (IntelliKorea, Seoul, Korea) and were saved as a STereoLithography (STL) file. Then, STL files were sliced in the *Z* direction. Finally, slices were projected by the projector for every layer to create the designed 3D morphology. Prints were carried out by repeating the process of projecting an image into the hydrogel followed by raising the Z-stage. Printing parameters were used as follows: printing thickness, 50 μm; number of base layer, 3; curing time of base layer, 4 s; number of buffer layer, 1; and curing time of buffer layer, 3 s. Totally, we printed 40–200 layers depending on test type. After printing, the printed object was briefly rinsed with deionized water to remove unreacted solution.

### Physical properties

Specimens with 1 × 1 × 0.2-mm^3^ rectangular shape were printed for water uptake ability measurement^60^. Briefly, samples were hydrated for 0.3, 0.5, 1, 2, 3, 4, and 5 h in 1× PBS (pH 7.4) at 37 °C. PBS was completely removed at each sampling time and the gels were weighed to obtain *W*_swollen_. Then the gels were lyophilized and measured the dried weight (*W*_dry_). The ratio of water uptake from dried Sil-MA (*Q*) can be calculated as following equation

$${Q} = \left( {{W}_{{\mathrm{swollen}}}-{W}_{{\mathrm{dry}}}} \right){/W}_{{\mathrm{dry}}} \times 100\left( \% \right)$$

Expansion rate from DLP product by water, PBS, and medium was estimated by measuring the length of *X*- and *Y*-axis based on initial status right after printing. Disc-shaped specimens with 2.5 cm in diameter and 2 mm thickness were printed for rheological analysis. The rheological properties of Sil-MA were measured at 25 °C using an Anton Paar MCR 302 (Anton Paar, Zofingen, Switzerland) rheometer. Peltier element and a thermostatic hood for temperature control were equipped in the rheometer and a UV source (SP-LED-1, 365 nm, USHIO, Cypress, CA) was added for photocrosslinking to optimally prepare the hydrogel prepolymer. The measurements were performed utilizing a sandblasted-type geometry (PP25 s^−1^, 25 mm in diameter) with a humidified atmosphere in the measuring chamber. The edge of the specimen and surfaces were covered with oil to avoid sample drying. Minimum torque oscillation was 0.5 nN m and maximum torque was 200 mN m.

The dynamic storage (*G*′) and loss modulus (*G*″) of materials were measured in oscillatory mode at 25 °C. In the amplitude sweep measurement, various amounts of Sil-MA with LAP (0.2% w/v) were tested for strain value of 0.01 to 1% by keeping the frequency constant at 10 rad s^−1^. In case of the frequency sweep measurement, the hydrogel materials were tested from 0.158 to 25.1 rad s^−1^ frequency range by keeping the shear strain constant at 1%. For the UV-curing test depending on concentration of Sil-MA and LAP, oscillatory measurements were performed at a frequency of 1 rad s^−1^ and the target strain value of 0.1%, which was found to be in the linear viscoelastic region for both the liquid and the gelled state of Sil-MA. The crossover points of *G*′ and *G*″ were defined as the gelation points. UV light shined on the specimen on the glass plate (CTD600/UV) for 300 s.

The compressive stress–strain curve of hydrogel was obtained by applying uniaxial compression force with a universal testing machine (QM100S, QMESYS, Gunpo, Korea) that was equipped with a 10 kgf load cell in an unconfined environment (Supplementary Fig. 2a). Hydrogel disks with dimensions of 11.2 mm (diameter) and 10 mm (height) were studied in the compressive tests. A compression force was loaded at a displacement rate of 5 mm min^−1^ until the specimen broke to calculate the stress at failure and strain at failure. The tensile test was carried out on QMESYS with tensile jigs at a stretch velocity or 5 mm min^−1^ at room temperature in air, using a hydrogel specimen cut into the dumbbell shape of a concave column with measurements the size of length 16 mm (*L*) and width 7 mm (*W*) (Supplementary Fig. 2b). The sample thickness was 2 mm. All specimens for mechanical testing were prepared via DLP printing. From the stress–strain curve, the secant modulus at 1, 25, and 50% strain for compressive test and at 50% for tensile test was calculated. All tests of physical properties were performed at strictly controlled condition room (80% humidification, 27 °C) to prevent drying out.

### Hydrogel suturing and ex vivo study

A hydrogel membrane of 2.5 cm × 2.5 cm × 0.15 cm (*W* × *L* × *H*) was printed out through DLP printer. This membrane was rolled out and sutured end to end. For ex vivo study, larynx and trachea from male beagle dog (15 kg) was fixed with 4% formalin, which was donated from the animal center of Kangwon University (Korea). Trachea end to end anastomosis of larynx and trachea was carried out using DLP-printed Sil-MA trachea (2.5 cm × 2 cm × 1 cm: *W *× *L*× *H*). To suture hydrogel itself and between trachea and hydrogel, simple interrupted suture was applied using 3-0 coated VICRYL^®^ (Ethicon, Johnson & Johnson, USA).

### Printability

Porous scaffolds and Eiffel Tower models with complex detail were printed by using 30%Sil-MA (424 mM GMA) solution consisted with LAP (0.2% w/v) through DLP printer. In addition, various organ shapes such as ear, brain, trachea, heart, lung, and blood vessel network were printed by using the same 30%Sil-MA bioink. After printing, the newly created ear and brain were pressed by an adult male’s fingers (30 years old). To visualize the mechanical distortion of the 30%Sil-MA hydrogel-printed organs, compressive test was performed using a 7 kg kettle bell for 3 min.

### The actual resolution and accuracy of 3D DLP printing using Sil-MA

In order to check the actual resolution and similarity to the CAD files of the developed system, 30%Sil-MA was 3D-printed into a block with gradually degrading circle or square patterns ranging from 900 to 70 µm in the planar axis and from 900 to 200 µm in the vertical axis. The actual figure size was measured and the deviation was compared to the nominal sizes in both the horizontal and vertical planes.

### Cell viability and proliferation

NIH/3T3 mouse fibroblast cells were purchased from Koram Biotech (ATCC distributor, Korea). Chondrocytes were isolated from human septal cartilage obtained from patients who underwent septoplasty, with informed consent. The procedures described in this study were approved by the Institutional Review Board of Chuncheon Sacred Heart Hospital of Hallym University (IRB No. 2017–87). The cartilage was washed several times with sterile PBS and chopped as finely as possible using a scalpel blade under aseptic conditions. Then the minced samples digested with 0.6% collagenase A (Roche Applied Science, Germany) at 37 °C for 6 h. Isolated chondrocytes were cultured into 10 cm culture dishes until confluent and sub-cultured up to two passages. The cell medium consists of Dulbecco’s modified Eagle medium with 10% v/v fetal bovine serum and 1% v/v penicillin-streptomycin. All cultures were maintained in 5% CO_2_ incubator at 37 °C with the medium changed every 3 days. Cells are detached using trypsin-EDTA 0.25%, counted, and prepared for the DLP printing. In all, 1 × 10^6^ cells were mixed with Sil-MA solution containing LAP (0.2% w/v). Afterwards, this cell-loaded Sil-MA solution was used as bioink for DLP printing. The printed architectures were cultured for 14 days in the above culture medium. Cytotoxicity was evaluated using a Live/dead assay kit (Life Technologies, USA) according to the manufacturer’s instruction. Samples were visualized and imaged using a fluorescent microscope (Eclipse 80i, Nikon, Tokyo, Japan) after 24 h incubation at 37 °C and 5% CO_2_. Cell proliferation in the cell-laden hydrogel was examined by CCK-8 assay (Dojindo Molecular Technology, Rockville, USA) according to the manufacturer’s protocol.

### Cell distribution in Sil-MA hydrogel

For live-cell imaging, NIH/3T3 cell line was stained with PKH26 and PKH67 (red and green fluorescence, Sigma-Aldrich) as the manufacturer’s instruction. Cells were mixed with Sil-MA solution at a density of 2 × 10^7^ cells per mL for red labeling and 1 × 10^7^ cells per mL for green labeling. Samples (4 × 4 × 4 mm^3^), including a logo (Hallym University), a trachea, and brain were printed by DLP printer. Light sheet microscopy referred to as single plane illumination microscopy (SPIM) allowed to be obtained multidimensional imaging to be performed from several microns to several millimeters, minimizing photobleaching and phototoxic effect and enhancing image contrast and axial resolution. The cell distribution in the 30%Sil-MA hydrogel was detected using Lightsheet Z.1 microscope (Carl Zeiss Microscopy GmbH, Jena, Germany) with Lightsheet Z.1 detection optics ×5/0.16 and two-sided Illumination Optics Lightsheet Z.1 ×5/0.1 equipped with two detection module PCO at KBSI (Chuncheon, Korea). Edge cameras (sCMOS sensor, pixel size of 6.5 μm, 1920 × 1920 pixel resolution, 15-bit depth, 30 fps at 1000 × 1000 pixel). These samples were glued on the sample chamber in front of the detection lens. It was possible to rotate the sample and collect 3D stacks from multiple angles. One of the printed logos was observed by confocal microscope (Nanoscope, Korea).

### Cartilage tissue engineering

30%Sil-MA solution containing 1 × 10^7^ cells per mL of human chondrocytes was printed out to a trachea-shaped ring-like cartilage (7 mm of external diameter, 5 mm of internal diameter, and 6 mm of height) by DLP printer for cartilage tissue engineering. For in vitro test, the cell-loaded Sil-MA hydrogels were kept in a standard CO_2_ incubator for 4 weeks. All constructs were evaluated at each time point of 1, 2, 3, and 4 weeks.

### Histological evaluation

Samples from in vitro tests were fixed with 10% formaline for 24 h, immersed in 30% sucrose for 1 day and molded with Tissue-Teck Optimum Cutting Temperature compound (Tissue Tek, Elkhart, IN, USA), and frozen in liquid nitrogen. Frozen samples were cut to a 10 μm thickness, and encapsulated chondrocytes, general structure of tissue, and chondrogenesis were examined. To see the PKH26-labeled and encapsulated cells in hydrogel, sections were stained with Dapi (Vector Lab., Burlingame, USA) and observed under a fluorescence microscope (Eclipse 80i, Nikon Co., Japan). H&E (HE) to assess tissue morphology, Safranin-O staining to identify the presence of proteoglycan-rich matrix, Masson’s Trichrome (MT) staining to detect to collagen production were applied. Stained sections were analyzed under a microscope (Eclipse 80i, Nikon Co., Japan).

### Degradation study

From above samples, 10 μm-thickness sections were prepared without staining. The autofluorescence from SF was taken by a confocal imaging plate reader, InCell^®^ analyzer 2200 (GE Healthcare, Piscataway, USA) with a 475 nm excitation line and 525 nm band pass filter. The signal strength from the Sil-MA autofluorescence was measured by InCell^®^ developer toolbox after stitching pictures using InCell^®^ analyzer workstation. The remaining Sil-MA was assessed as a percentage of the Sil-MA area at each time point versus the initial area of the Sil-MA hydrogel^61^.

### Statistical analysis

Student unpaired (two-sample) *t*-test was used for statistical analysis. Data were presented as the mean value ± standard deviation.

### Data availability

The authors declare that all data supporting the findings of this study are available within the article and its Supplementary Information files or from the corresponding author on reasonable request.

## Electronic supplementary material

Supplementary Information

Description of Additional Supplementary Files

Supplementary Movie 1

Supplementary Movie 2

Supplementary Movie 3

Supplementary Movie 4

Supplementary Movie 5

Supplementary Movie 6

Supplementary Movie 7

Supplementary Movie 8
