# Publisher Correction: Precisely printable and biocompatible silk fibroin bioink for digital light processing 3D printing

**DOI:** 10.1038/s41467-018-04517-w

**Published:** 2018-06-11

**Authors:** Soon Hee Kim, Yeung Kyu Yeon, Jung Min Lee, Janet Ren Chao, Young Jin Lee, Ye Been Seo, Md. Tipu Sultan, Ok Joo Lee, Ji Seung Lee, Sung-il Yoon, In-Sun Hong, Gilson Khang, Sang Jin Lee, James J. Yoo, Chan Hum Park

**Affiliations:** 1 Nano-Bio Regenerative Medical Institute, College of Medicine, Hallym University, Chuncheon, 24252 Republic of Korea; 2 School of Medicine, George Washington University, Washington, DC 20037 USA; 3 Division of Biomedical Convergence, College of Biomedical Science, Kangwon National University, Chuncheon, 24341 Republic of Korea; 4 Department of Molecular Medicine, School of Medicine, Gachon University, Incheon, 406-840 Republic of Korea; 5 Department of BIN Convergence Technology, Department of Polymer Nano Science & Technology and Polymer Materials Fusion Research Center, Chonbuk National University, Jeonju, 54896 Republic of Korea; 6 Wake Forest Institute for Regenerative Medicine, Wake Forest School of Medicine, Medical Center Boulevard, Winston-Salem, NC 27157 USA; 7 Departments of Otorhinolaryngology-Head and Neck Surgery, Chuncheon Sacred Heart Hospital, School of Medicine, Hallym University, Chuncheon, 24252 Republic of Korea

Correction to: *Nature Communications* 10.1038/s41467-018-03759-y, published online 24 April 2018

The original version of this Article contained errors in Figs. 5 and 6. In Fig. 5b, the second panel on the bottom row was stretched out of proportion. The correct version of Fig. 5b appears below as Fig. 1:

**Fig. 1:**
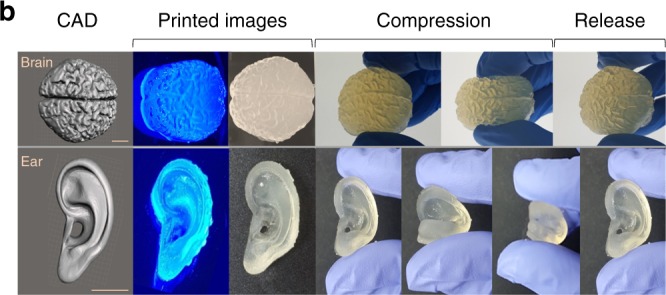
.

which replaces the previous incorrect version that appears as Fig. 2 below.

**Fig. 2:**
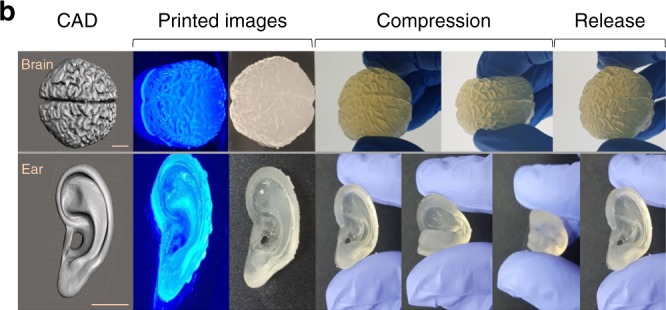
.

In Fig. 6d, the first panel was also stretched out of proportion. In Fig. 6f, the fifth panel inadvertently repeated the fourth. The correct version of Fig. 6c–f which appears below as Fig. 3:

**Fig. 3:**
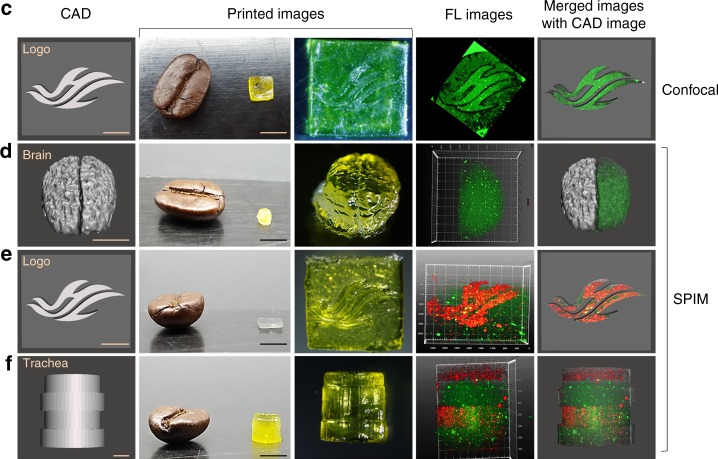
.

which replaces the previous incorrect version that appears as Fig. 4 below.

This has been corrected in both the PDF and HTML versions of the Article.

**Fig. 4:**
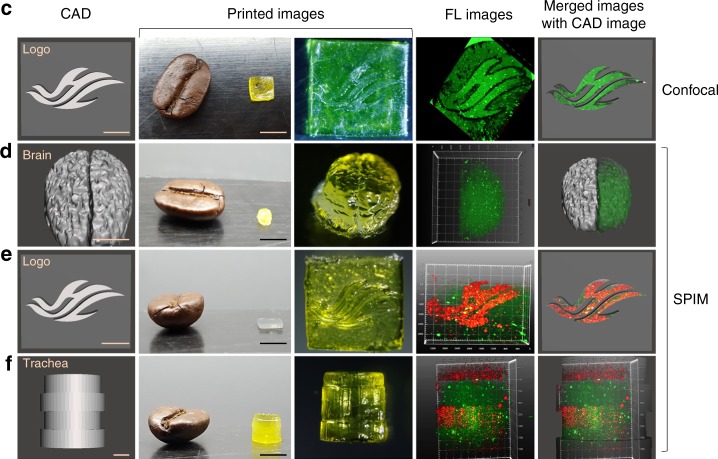
.

