# Supplementary material for: Precisely printable and biocompatible silk fibroin bioink for digital light processing 3D printing
Source: Nat Commun. 2018 Apr 24;9:1620. doi: 10.1038/s41467-018-03759-y (PMC5915392; doi:10.1038/s41467-018-03759-y)
Supplement: Supplementary file 1 — Supplementary Information [file 41467_2018_3759_MOESM1_ESM.pdf]

<u>Supplementary Information</u>

for "Precisely printable and biocompatible silk fibroin bio-ink for digital light processing 3D printing"

Kim *et al.*

Sil-MA Contents (%)	Phase Shift Angle (°)	Storage Modulus, G' (Pa)	Loss Modulus, G" (Pa)
**10**	6.38	81.1	9.1
**20**	6.51	1,068.2	121.9
**30**	9.07	3,038.3	485.1

Supplementary Table 1. Rheological analysis of Sil-MA hydrogel depending on Sil-MA contents at frequency 10 Hz and strain 1%

**LAP contents (%) (at 10%Sil-MA)**	**0.05**	**0.1**	**0.2**	**0.4**
Gel point (s)	101	83	74	41
**Sil-MA contents (%) (at LAP 0.2%)**	**10**	**20**	**30**	
Gel point (s)	74	81.2	137.5	

Supplementary Table 2. Gel point depending on LAP and Sil-MA contents

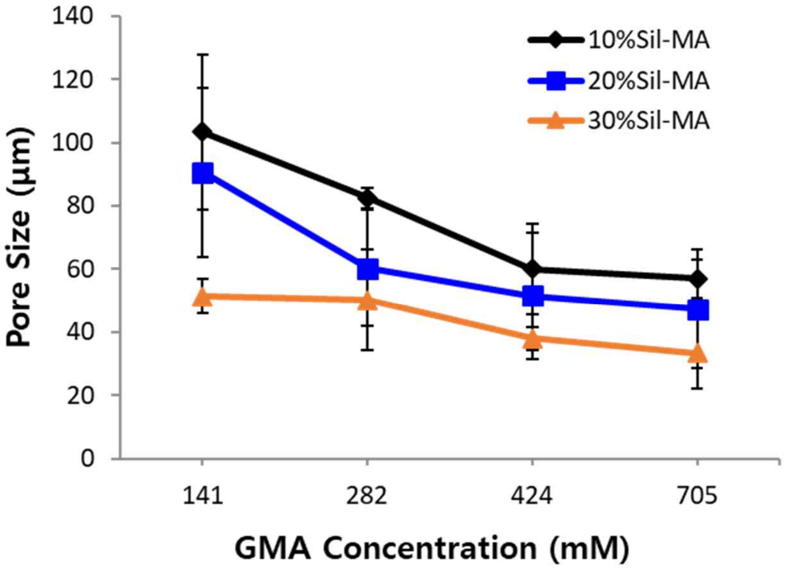

Supplementary Figure 1. Pore size depending on glycidyl methacrylate (GMA) concentrations in hydrogel. The pore size showed decreasing pattern with higher degrees of methacrylation and concentration of silk fibroin (SF) modified by GMA (Sil-MA). Data are presented as mean±s.d. Each assay was conducted in triplicate.

a.

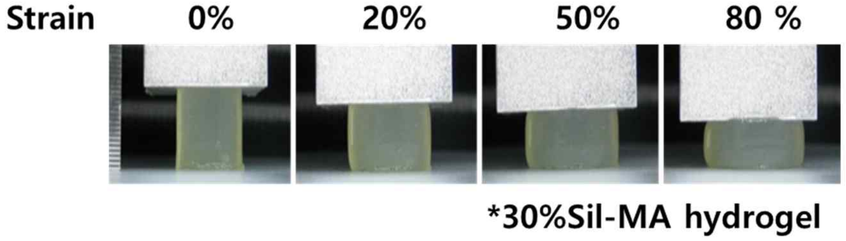

b.

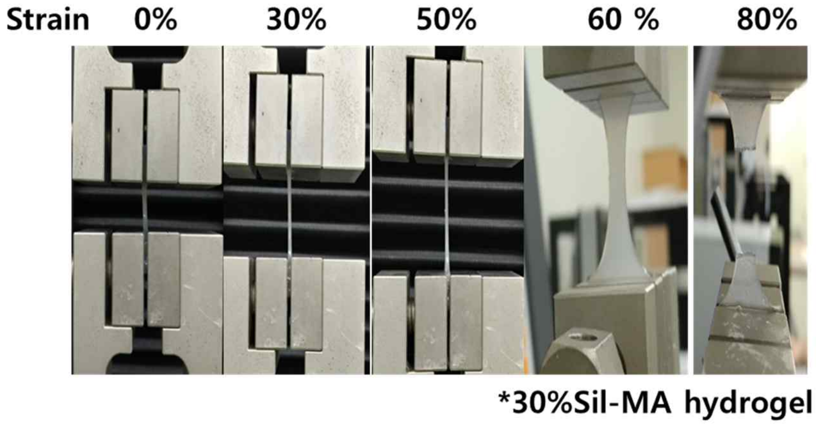

Supplementary Figure 2. The process of mechanical tests of Sil-MA hydrogel. Samples were fabricated by the Digital Light Processing (DLP) manufacturing machine. **(a)** The compressive stress-strain curve of hydrogel was obtained by applying uniaxial compression force with a Universal Testing Machine (UTM) that was equipped with a 10 kgf load cell in an unconfined environment. Hydrogel disks with dimensions of 11.2 mm (diameter) and 10 mm (height) were studied in the compressive tests. A compression force was loaded at a displacement rate of 5 $mm \cdot min^{-1}$ until the specimen broke to calculate the stress-at-failure and strain-at-failure. **(b)** The tensile test was carried out on with tensile jigs at a stretch velocity or 5 $mm \cdot min^{-1}$ at room temperature in air, using a hydrogel specimen cut into the dumbbell-shape of a concave column with measurements the size of length 16 mm (L) and width 7 mm (W). The sample thickness was 2 mm.

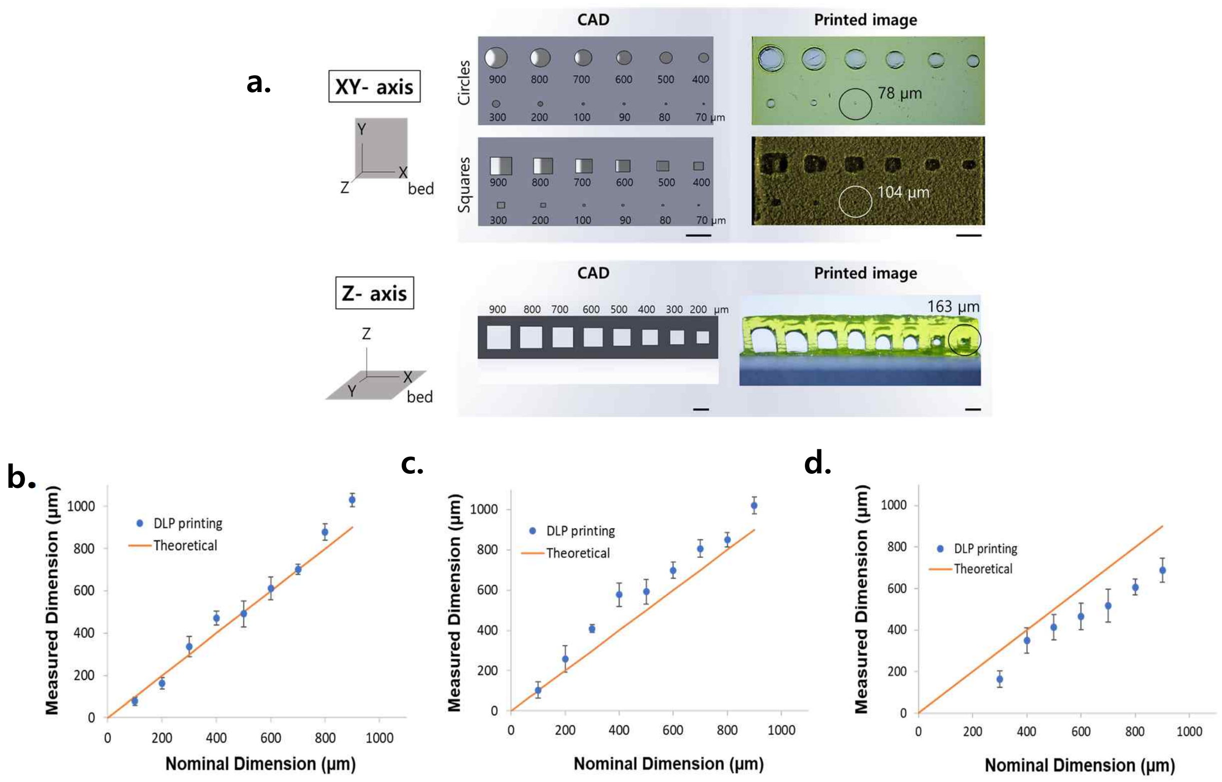

Supplementary Figure 3. **Resolution and accuracy testing of DLP printing using Sil-MA in three axes (X, Y and Z) (a)** CAD drawing and printed images of the standard structures. Round marks indicate the smallest hole printed. Scale bar indicates 1 mm. (**b-d**) Plots of the measured circle diameter and rectangular width against nominal dimensions. The measured (**b**) circle sizes and rectangular sizes **(c)** in the horizontal direction and (**d**) in the vertical direction (theoretical graph slope = 1 for comparison between measured). Data are presented as mean±s.d. Each assay was conducted in triplicate. / The ultimate XY resolution is obtained by the DMD resolution (pixel). The Z resolution presented by the manufacturer is the minimum thickness of the resin layer created by the Z stepper motor moving the build plate [Ref 1]. However, the actual output is determined by both the optical resolution and the material properties. Therefore, to check the actual resolution and accuracy of our DLP system using 30%Sil-MA, standard structures containing circles and rectangles 70 to 900 µm in size were produced in parallel with the build plate. In addition, to determine Z resolution, a standard structure containing circles 200 to 900 µm in size was produced perpendicular to the build plate. Finally, we measured the actual figure size and determined the deviation by comparing the nominal diameters in both the horizontal and vertical planes. The observable features were formed when nominal X and Z dimensions were at least 100 and 300 µm, respectively. Our system produced spatial accuracy with an average deviation of 66 µm (circle diameter), 90 µm (square width) and 142 µm (height).

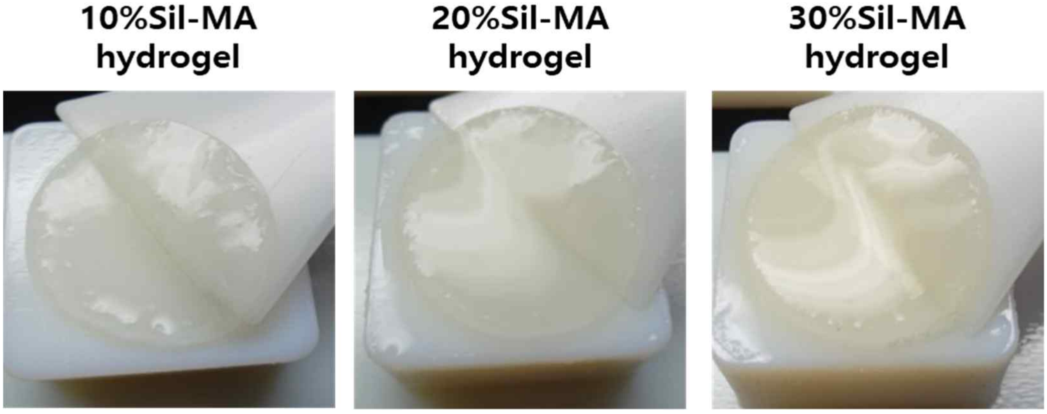

Supplementary Figure 4. Handling of Sil-MA hydrogel after DLP printing. The 30%Sil-MA was selected for the printability test because it printed as designed for both pattern and size as well as ease of handling after DLP printing.

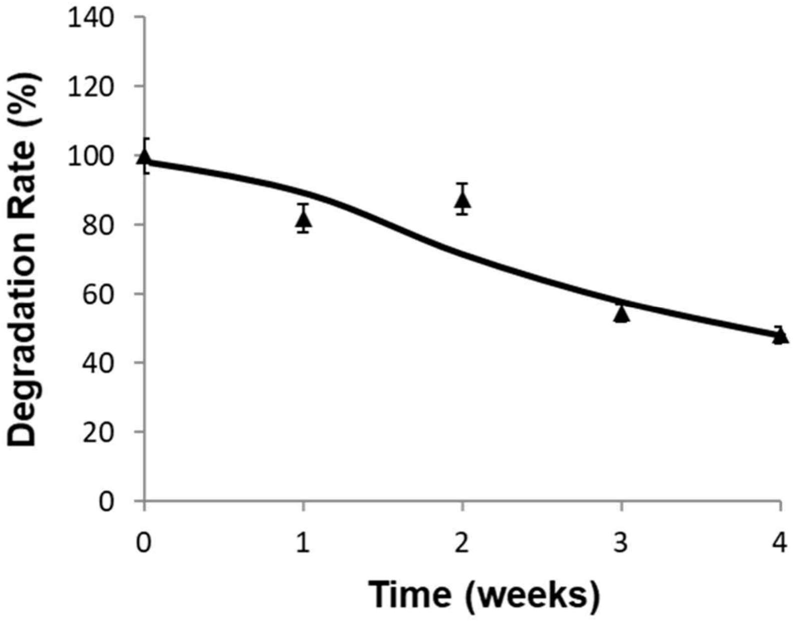

Supplementary Figure 5. *In vitro* degradation profile of Sil-MA hydrogel with human chondrocytes. Biodegradability is one of the important factors of biomaterial for use in tissue engineering because the supportive structures exert a strong influence on cell migration, proliferation, differentiation, and the shape of the formed tissue. Commonly, hydrogel has a faster degradation rate than other forms and has weak mechanical strength which can lead to its collapse and inadequate tissue formation. One goal of 3D printing is creation of cartilaginous structures that require slow degradation of the scaffold, allowing enough time for tissue regeneration. Here, we evaluated degradation rate of 30%Sil-MA hydrogel with its potential value for DLP printing. This graph shows degradability of 30%Sil-MA hydrogel with cells *in vitro*. Cell loaded Sil-MA hydrogel degraded gradually and exhibited a 50% degradation rate at 4 weeks after cultivation. Data are presented as mean±s.d. Each assay was conducted in triplicate.

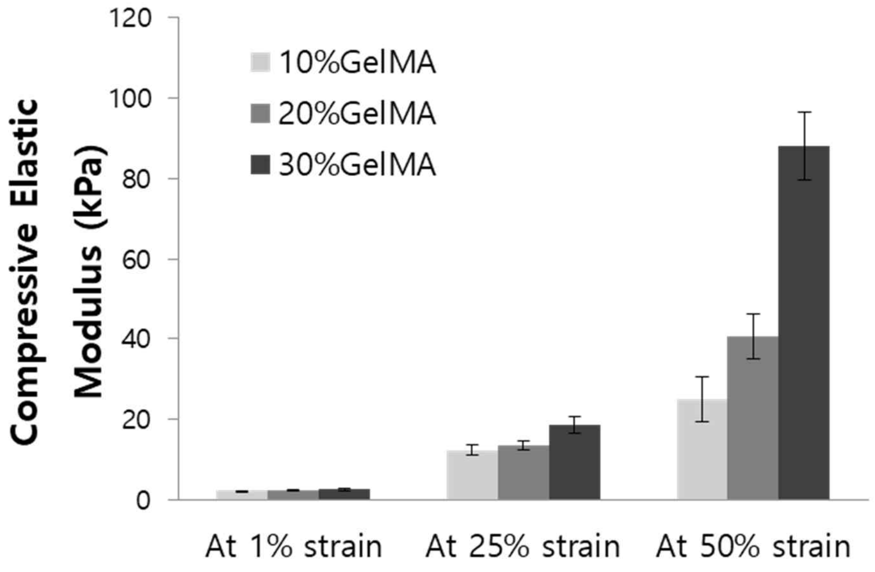

Supplementary Figure 6. Secant modulus of GelMA hydrogel at 1%, 25% and 50% strain. The compressive elastic modulus of 30%Sil-MA was about 1.5 times higher than that of 30%Gel-MA (88 kPa). Each assay was conducted in triplicate.

Supplementary Reference

1. Urrios, A.*, et al.* 3D-printing of transparent bio-microfluidic devices in PEG-DA. *Lab on a Chip* **16**, 2287-2294 (2016).
