# Supplementary material for: Precisely printable and biocompatible silk fibroin bioink for digital light processing 3D printing
Source: Nat Commun. 2018 Apr 24;9:1620. doi: 10.1038/s41467-018-03759-y (PMC5915392; doi:10.1038/s41467-018-03759-y)
Supplement: Supplementary file 2 — Description of Additional Supplementary Files [file 41467_2018_3759_MOESM2_ESM.pdf]

Description of Additional Supplementary Files

for "Precisely printable and biocompatible silk fibroin bio-ink for digital light processing 3D printing"

Kim *et al.*

File name: Supplementary Movie 1

Description: Supplementary Movie 1 shows the suturing process of Sil-MA hydrogel sheet.

File name: Supplementary Movie 2

Description: Supplementary Movie 2 shows the process of end to end anastomosis of dog's trachea using Sil-MA hydrogel ring.

File name: Supplementary Movie 3

Description: Supplementary Movie 3 shows the process of pressing and releasing the brain shaped Sil-MA hydrogel with fingers.

File name: Supplementary Movie 4

Description: Supplementary Movie 4 shows the process of pressing and releasing the ear shaped Sil-MA hydrogel with fingers.

File name: Supplementary Movie 5

Description: Supplementary Movie 5 taken by confocal microscope shows Sil-MA hydrogel printed out to be the letter HL(the logo of Hallym University). Green signal indicates cells labeled with PKH67 encapsulated in Sil-MA hydrogel.

File name: Supplementary Movie 6

Description: Supplementary Movie 6 taken by single plane illumination microscopy (SPIM) which rotates 360 degrees shows Sil-MA hydrogel printed out to be the shape of human brain. Green signal indicates cells labeled with PKH67 encapsulated in Sil-MA hydrogel.

File name: Supplementary Movie 7

Description: Supplementary Movie 7 taken by SPIM which rotates 360 degrees shows Silk-MA hydrogel printed out to be the letter HL. The printed letter consists of two different layers -PKH67 labeled cells (green) and PKH26 labeled cells (red).

File name: Supplementary Movie 8

Description: Supplementary Movie 8 taken by SPIM which rotates 360 degrees shows Sil-MA hydrogel printed out to be the shape of 4 cylinders piled up to be the shape of winding trachea; green and red cells by turns. This trachea shape consists of PKH67 labeled cells (green) and PKH26 labeled cells (red).
